# Bioremoval of the synthetic dye malachite green by marine *Trichoderma* sp

**DOI:** 10.1186/2193-1801-3-631

**Published:** 2014-10-25

**Authors:** Kandasamy Saravanakumar, Kandasamy Kathiresan

**Affiliations:** Centre of Advanced Study in Marine Biology, Faculty of Marine Sciences, Annamalai University, Parangipettai, 608502 Tamil Nadu India

**Keywords:** Marine *Trichoderma*, *Hypocrea lixii*, Mangroves, Malachite green, FTIR, SEM

## Abstract

In the present study, a marine strain of *Trichoderma* sp was used for degradation of a synthetic dye, malachite green. Individual and interaction effects of the physical and chemical factors that influenced the percentage of dye degradation were tested by response surface methodology. For optimization, enzyme production and dye degradation were assessed under different temperatures (5–40°C), pH values (3–11), yeast extract (5–9 g L^-1^) and incubation period (0–15 days). The optimum conditions found for dye degradation, were 30°C, pH 5.8, 5.81 mg L^-1^ yeast extract for an incubation period of 10 days. Whereas for laccase production they were 29°C, pH 5.3, 7.7 mg L^-1^ yeast extract for an incubation period of 12 days. It was confirmed that laccase production required the higher nitrogen source. Degradation of dye was confirmed by using analytical techniques such as FTIR, UV–vis spectral and scanning electron microscope analysis. Furthermore, toxicity effect of degraded and undegraded dye solutions was tested with *Artemia salina*. Hundred percent mortality was observed in undegraded dye solution as against only 2-5% in degraded dye solution. This work proved the potential of marine strain of *Trichoderma Hypocrea lixii* on dye degradation.

## Introduction

Textile synthetic dyes have stable and toxic molecules. They affect biological systems by inducing mutations and causing cancer, when they are discharged in to the water bodies. Many methods are used for dye removal, they includephysical/chemical adsorption, oxidation, biological treatments (Akar et al. 2013), microbial biomass and enzyme treatments (Anjaneyulu et al. 2005). Among these, biological treatment methods especially using microbial enzymes are highly efficient for dye degradation (Peralta-Hernandez et al. 2009; Baldeva et al. 2013). Azo/synthetic dyes contain aromatic and phenolic compounds. Degradation of these compounds in azo dyes is a challenge in current research. Microbial enzymes are capable of removing phenolics and aromatic amines present in the azo dyes (Claus 2003; Casas et al. 2007). The enzyme laccase, has enormous biotechnological applications such as in paper pulp bleaching, decolorization of synthetic dyes, wine clarification, fruit juice processing, bioremediation, ethanol production, biosensors, biofuel cells, organic synthesis and drug synthesis (Mayer and Staples 2002; Hadzhiyska et al. 2006; Zhu et al. 2011). Generally industrial dyes are highly tolerant to light, temperature, oxidization. Hence enzymatic degradation is preferable over physicochemical methods of dye degradation (Niladevi and Prema 2008). Microbial enzymes have been studied mostly for terrestrial fungal strains in dye degradation (Shubo Deng et al. 2005). They are economical and can be applied to a wide range of dyes degradation process (McMullan, et al. 2001; Robinson et al. 2001; Saparrat and Hammer 2006; Xiangkang et al. 2011). Such a study in marine strains is largely wanting. Hence the present study was undertaken with the objectives of optimizing conditions required for the laccase production by *T. harzianum/Hypocrea lixii* TSK8 and also enzymatic degradation of synthetic dye by using statistical method.

## Materials and methods

### Chemicals

All the chemicals including Malachite Green were purchased from Himedia, Merck and Sigma, India.

### Preparation of dye solution

Stock solution of Malachite Green was prepared by dissolving the dye in distilled water to concentrations of 100–250 mgL^-1^. 250 mg L^-1^ of dye solution is equivalent to 100% of Malachite Green.

### Culture conditions and culture identification

The fungus *T. harzianum/Hypocrealixii* TSK8 (JQ809340) isolated from mangroves was used for this experiment. The identity of the fungal strain was confirmed based on 18S rRNA sequence in comparison with that available in NCBI database by using BLAST methods. This strain was stored on potato dextrose agar (PDA) slants at 4°C for the further experiments (Saravanakumar et al. 2013).

### Laccase production and dye degradation

Laccase production and dye degradation experiments were carried out in 30 runs using central composite design of the response surface methodology. This experimental design is presented in Table 1 along with experimental and predicted values of response. All these conditions of the experiment were carried out in 500 mL Erlenmeyer flasks. Each flask contained 100 mL of a medium consisted of MgSO_4_ (0.1 g), (NH_4_)_2_SO_4_ (0.6 g), NaCl (0.5 g), K_2_HPO_4_ (1.36 g), CaCl_2_ (0.02 g), MnSO_4_ (1.1 g), ZnSO_4_ (0.2 g), CuSO_4_ (0.2 g) and FeSO_4_ (0.14 g) in 1000 mL of 50% seawater and 750 ppm of malachite green. The fungal strain of *Hypocrea* was cultivated in the potato dextrose agar medium in a Petri plate at 30°C for 5–10 days. The agar plugs of 8 mm diameter were cut and used as inoculum for laccase production and dye degradation under different temperatures (5–40°C), pH levels (3, 5, 7, 9 and 11), yeast extract (5–9 gL^-1^) and incubation period (0–15 days). The quadratic model equation (Box and Behnken 1960) was used to evaluate the interaction and individual relationships of the factors on the responses of (Y1) % of dye decolouration and (Y2) laccase production (UmL^-1^) and the independent variables: X1 (temperature), X2 (pH), X3 (yeast extract) and X4 (incubation period).

**Table 1:** **Experimental design (CCD) of response surface methodology**

Std runs	(A) temperature (°C)	(B) pH	(C) yeast extract (g L ^-1^)	(D) incubation period (days)	% of dye degradation		Laccase production (UmL ^-1^)	
					Experimental	Predicted	Experimental	Predicted
1.0	10.0	3.0	5.0	0.0	0.20	0.82	0.02	0.22
2.0	40.0	3.0	5.0	0.0	1.50	2.61	0.03	0.11
3.0	10.0	11.0	5.0	0.0	1.60	2.23	0.02	0.14
4.0	40.0	11.0	5.0	0.0	1.20	4.43	0.01	0.22
5.0	10.0	3.0	9.0	0.0	0.20	0.44	0.20	0.15
6.0	40.0	3.0	9.0	0.0	1.20	2.77	0.32	0.05
7.0	10.0	11.0	9.0	0.0	1.20	2.14	0.01	0.05
8.0	40.0	11.0	9.0	0.0	3.50	4.87	0.02	0.66
9.0	10.0	3.0	5.0	15.0	87.71	93.26	2.71	2.85
10.0	40.0	3.0	5.0	15.0	87.01	94.76	2.01	2.23
11.0	10.0	11.0	5.0	15.0	87.74	94.87	2.74	2.97
12.0	40.0	11.0	5.0	15.0	86.80	96.78	1.80	2.76
13.0	10.0	3.0	9.0	15.0	87.15	92.61	2.15	2.20
14.0	40.0	3.0	9.0	15.0	86.45	94.64	1.45	2.11
15.0	10.0	11.0	9.0	15.0	86.70	94.50	1.70	2.61
16.0	40.0	11.0	9.0	15.0	87.87	96.94	2.87	2.92
17.0	5.0	7.0	7.0	7.5	88.39	78.25	3.39	2.92
18.0	55.0	7.0	7.0	7.5	88.45	72.64	3.45	2.58
19.0	25.0	1.0	7.0	7.5	86.54	76.42	1.54	1.53
20.0	25.0	15.0	7.0	7.5	87.17	72.55	2.17	1.10
21.0	25.0	7.0	3.0	7.5	88.99	78.89	3.99	3.51
22.0	25.0	7.0	11.0	7.5	88.89	78.67	3.89	3.29
23.0	25.0	7.0	7.0	7.5	87.55	86.98	2.55	3.19
24.0	25.0	7.0	7.0	22.5	87.24	66.93	2.24	1.16
25.0	25.0	7.0	7.0	7.5	88.90	86.98	3.90	3.19
26.0	25.0	7.0	7.0	7.5	88.90	86.98	3.90	3.19
27.0	25.0	7.0	7.0	7.5	88.90	86.98	3.90	3.19
28.0	25.0	7.0	7.0	7.5	88.90	86.98	3.90	3.19
29.0	25.0	7.0	7.0	7.5	88.90	86.98	3.90	3.19
30.0	25.0	7.0	7.0	7.5	86.74	86.98	1.74	3.19

### Statistical optimization of the experimental conditions

The data obtained from RSM on dye degradation and enzyme production were subjected to analysis of variance (ANOVA). The experimental results of RSM were fit via the response surface regression procedure, by following second order polynomial equation.
1

Where Yi is the predicted response, X_i_X_j_ are independent variables, β_0_ is the offset term, β_i_ is the i^th^ linear coefficient, βii is the i^th^ quadratic coefficient, and β_ij_ is the ij^th^ interaction coefficient. However, in this experiment, the independent variables were coded as X_1_, X_2_, X_3_ and X_4_. Thus, the second order polynomial equation can be presented as follows.
23

Whereas: X_1_ is temperature (10–40°C), X_2_ is pH levels (3, 5, 7, 9 and 11), X_3_ is yeast extract (5–9 gL^-1^), and X_4_ is incubation period (0-15days).

Statistical software namely the Design expert (8.0.6 package) was used for the regression analysis and to plot the response surface graphs of the experimental data.

### Analysis of dye decolouration and degradation

Five mL of culture filtrate from each group of flasks was drawn and centrifuged at 3,000 rpm for 60 min at 4°C temperature. After centrifugation, the supernatant was collected and the absorbance was determined at 640 nm using a spectrophotometer (Elico, Model 301). This was done according to the experimental setup derived from central composite design of response surface methodology. The change of absorbance value was converted into concentration of dye value for determining the degradation of Malachite Green dye. The percentage of decolouration was calculated according to the following formula (Ayed et al. 2010, 2011).


Where

*I* was the initial absorbance and

*F* the absorbance at incubation time *t*

### FT-IR analysis

Fourier Transform Infrared (FT-IR) spectroscopy was performed for testing the degradation products present in the degraded dye samples. For FT-IR analysis, biological treatment process was performed with 250 mL solution containing 25 mgL^-1^ of malachite green and 5 mL of *Hypocrea lixii* biomass. At the reaction times of 0 h (control) and 24 days, samples were drawn to get degradation products, extracted in 30 mL of diethyl ether, crystallized and then used for FTIR analysis (Ayed et al. 2010).

### Enzyme assays

Laccase activity was determined in a reaction mixture (2.0 mL) containing 1.7 mL sodium acetate buffer (20 mM, pH 4.0) at room temperature (25°C), then observed at 420 nm. The reaction was started by adding 0.2 mL of laccase solution (Telke et al. 2009). Laccase activity (UmL^-1^) was defined as the amount of enzyme requiredto oxidize 1 μM of *o*-tolidine per minute. Protein concentration was measured by the Lowry method (Lowry et al. 1951) using bovine serum albumin as the standard. All of the measurements were performed in triplicate.

### Molecular weight determination of laccase

Purification of laccase was done by the method proposed by Zhang et al. (2010). The culture filtrate was fractionated by ammonium sulphate precipitation and dialyzed at 4°C in 20 mM sodium acetate buffer (pH 4.8). The purity and molecular mass of the purified laccase was determined by SDS-PAGE using a 5% stacking gel and a 12% resolving gel and this was followed by staining with Coomassie brilliant blue R-250.

### Acute toxicity test

The acute toxicity was tested according to Mathew’s method (Matthews 1995). The methodology of the toxicity test with *Artemia salina* consisted of exposing them at nauplii (Instar I) stage, hatched freshly in a saline solution (34 g L^-1^), to the different concentrations of the dye before and after biodegradation, for a period of 24 h at 25°C. The degraded and non degraded dye solution was tested in different concentrations of 100, 75, 50, 33.3, 25, 16.6, 4.2 and 2.08%. Negative controls were carried out in parallel using only a synthetic marine salt solution. After 24 h of incubation, the number of dead larvae was counted and calculated percentage of the mortality.

## Results

### Statistical optimization of the factors

**Figure 1:**
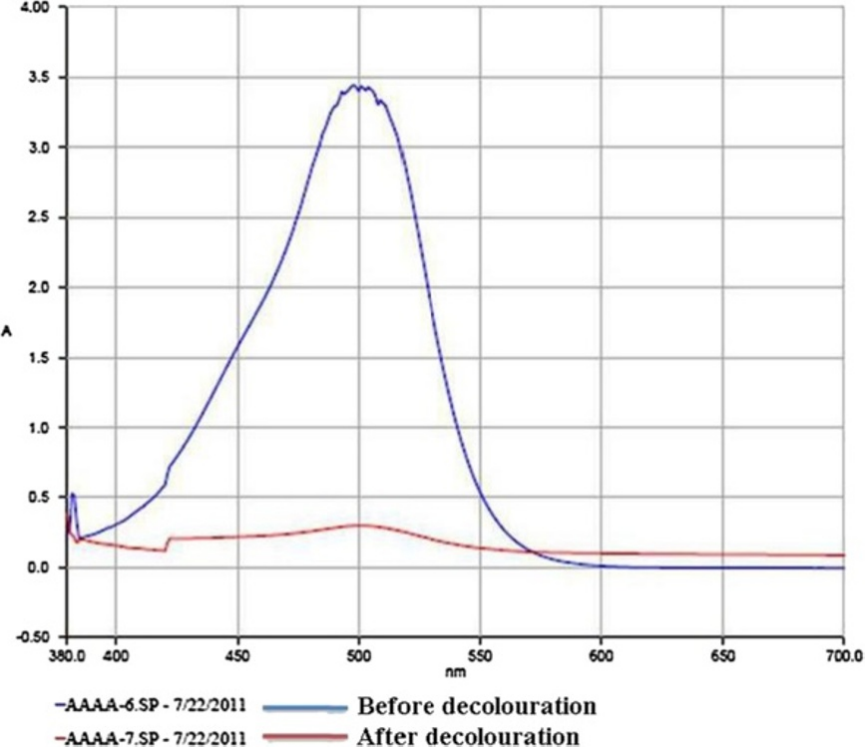
**Effects of Hypocrea lixii on dye decolouration (a) conical flasks; (b) UV- absorption spectrum.**

The effects of the different levels of the temperature, pH, yeast extract and incubation period on the dye degradation and laccase production by *Hypocrea lixii* was assessed by using a statistical model. The significant changes of dye decolouration are observed in Figure 1a and b.

### Analysis of model fitness

**Figure 2:**
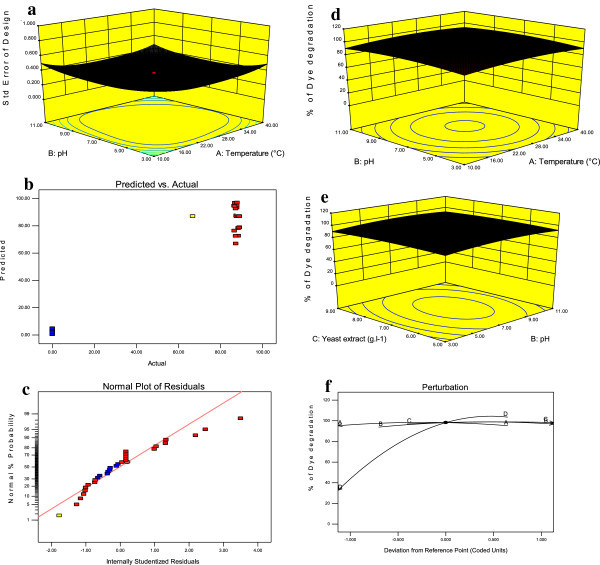
**Effect of culture conditions on dye degradation (a) standard error plot for the response of dye degradation and laccase production, (b) predicted and actual response of the dye degradation by** ***Hypocrea lixii*** **, (c) Normal probability, (d) significant interactive effect of pH and temperature, (e) significant effect of the yeast extract and pH, (f) optimization of the dye degradation of perturbation plot.**

The quadratic model fitness and acceptance was assessed based on the statistical significance and lack of fit on the response of dye degradation and laccase production. The probability values are shown in Tables 2 and 3 based on Student’s T- test and analysis of variance fitted to second order polynomial equation. The probability values of <0.05 indicated that the variables were statistically significant. The model was highly significant (F-20.09; P-0.0001; DF-14), and the lack of the fit was not significant (F-2.98; P-0.09; DF-9) for the response of dye degradation (Table 2). In the case of the laccase production the model was significant (F-2.62; P-0.03; DF-14) and also lack of fit was not significant (F-0.34; P-0.92; DF-9) and hence, the quadratic model was valid for the present study. A low value of standard error (0.43) between the measured and model data showed that the equation adequately represented actual relationship between dye degradation and laccase production (Figure 2a). High value of R^2^ of 0.94 and 0.70 was very close to the predicted value of R^2^ and it indicated a high dependence and correlation between the observed and the predicted values of response of dye degradation and laccase production respectively. The regression equation coefficients were calculated and the data were fit to a second-order polynomial equation. The response, dye degradation (Y1) and laccase production (Y2) by *Hypocrea lixii* is expressed in terms of the following regression equation:

**Table 2:** **Analysis of variance table (ANOVA) for response surface methodology of main effects and interacting effects of parameters in quadratic model for the dye degradation by** ***Hypocrea lixii***

Source	Sum of squares	df	Mean square	F value	p-value Prob > F
Model	42482.83	14	3034.488	20.09046	< 0.0001^***^
X_1_-Temperature (°C)	22.77126	1	22.77126	0.150762	0.7033^NS^
X_2_-pH	18.41882	1	18.41882	0.121946	0.007318^**^
X_3_-Yeast extract (g L^-1^)	0.069738	1	0.069738	0.000462	0.9831^NS^
X_4_-Incubation period (days)	36055.94	1	36055.94	238.7159	< 0.0001^***^
X_1_X_2_	0.16657	1	0.16657	0.001103	0.009739^**^
X_1_X_3_	0.278689	1	0.278689	0.001845	0.9663^NS^
X_1_X_4_	0.085404	1	0.085404	0.000565	0.9813^NS^
X_2_X_3_	0.081467	1	0.081467	0.000539	0.009818^**^
X_2_X_4_	0.040612	1	0.040612	0.000269	0.9871^NS^
X_3_X_4_	0.075826	1	0.075826	0.000502	0.9824^NS^
X_1_^2^	298.9977	1	298.9977	1.979577	0.1798^NS^
X_2_^2^	320.0991	1	320.0991	2.119283	0.1661^NS^
X_3_^2^	118.8562	1	118.8562	0.786912	0.3890^NS^
X_4_^2^	12614.69	1	12614.69	83.51817	< 0.0001^***^
Residual	2265.618	15	151.0412		
Lack of Fit	1851.681	9	205.7423	2.982222	0.0984^NS^
Pure Error	413.9376	6	68.9896		
Cor Total	44748.45	29			

Statistically significant ***(P <0.0001), **(P < 0.01), NS Non-significant.

**Table 3:** **Analysis of variance table (ANOVA) for response surface methodology of main effects and interacting effects of parameters in quadratic model for the laccase production by** ***Hypocrea lixii***

Source	Sum of squares	df	Mean	F value	p-value Prob > F
Model	48.56277	14	3.468769	2.621997	0.0371*
X_1_-Temperature (°C)	0.000179	1	0.000179	0.000136	0.0009***
X_2_-pH	0.717105	1	0.717105	0.54205	0.00729**
X_3_-Yeast extract (g L^-1^)	0.069738	1	0.069738	0.052714	0.8215^NS^
X_4_-Incubation period (days)	25.37625	1	25.37625	19.18158	0.0005***
X_1_X_2_	0.16657	1	0.16657	0.125908	0.7277^NS^
X_1_X_3_	0.278689	1	0.278689	0.210657	0.6528^NS^
X_1_X_4_	0.085404	1	0.085404	0.064556	0.8029 ^NS^
X_2_X_3_	0.081467	1	0.081467	0.06158	0.8074 ^NS^
X_2_X_4_	0.040612	1	0.040612	0.030698	0.8633 ^NS^
X_3_X_4_	0.075826	1	0.075826	0.057316	0.8140 ^NS^
X_1_^2^	0.395835	1	0.395835	0.299206	0.5924 ^NS^
X_2_^2^	7.268732	1	7.268732	5.494339	0.0333*
X_3_^2^	0.080502	1	0.080502	0.06085	0.8085 ^NS^
X_4_^2^	19.98253	1	19.98253	15.10453	0.0015**
Residual	19.84424	15	1.322949		
Lack of Fit	6.769842	9	0.752205	0.345196	0.9261^NS^
Pure Error	13.0744	6	2.179066		
Cor Total	68.40701	29			

Statistically significant ***(P <0.0001), **(P < 0.01), *(P < 0.05), NS Non-significant.

45

Whereas: X_1_ is temperature X_2_ is pH X_3_ is yeast extract and X_4_ is incubation period.

Further confirmation of the experimental model for the dye degradation was tested by plotting the normal probability and predicted and observed response of the dye degradation (Figure 2b and c). The regression analysis of the optimization study indicated that the model terms, X_2_, X_4_, X_4_^2^, X_1_X_2_ and X_2_X_3_ were significant on dye degradation by *Hypocrea lixii* (P <0.05) (Table 2 and Figure 2d and e). These results indicated that pH, incubation period and interactions of the factors such as temperature and pH had the direct association with dye degradation. Lack of fit (0.09) also suggested that the obtained experimental data were of a good fit with the model (Table 2). The optimal conditions for the maximum dye degradation were assessed by using the statistical perturbation plot (Figure 2f). The maximum dye degradation was observed under the optimal conditions: temperature 30°C, pH of 5.8, yeast extract 5.81 mg L^-1^ at incubation period of 10 days (Figure 2f).

**Figure 3:**
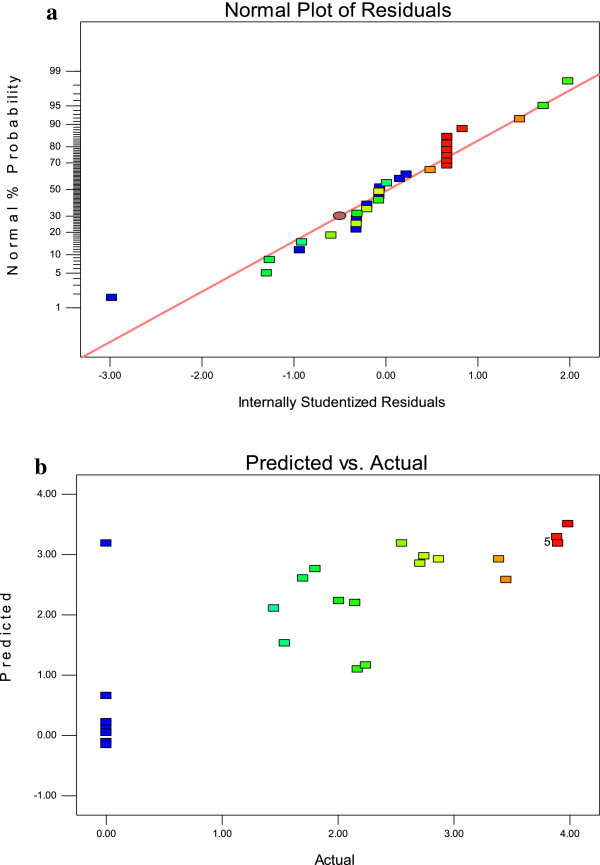
**Effect of the factors on laccase production by** ***Hypocrea lixii*** **(a) Normal probability plot for laccase production (b) Regression of predicted and actual response of laccase production by** ***Hypocrea lixii.***

**Figure 4:**
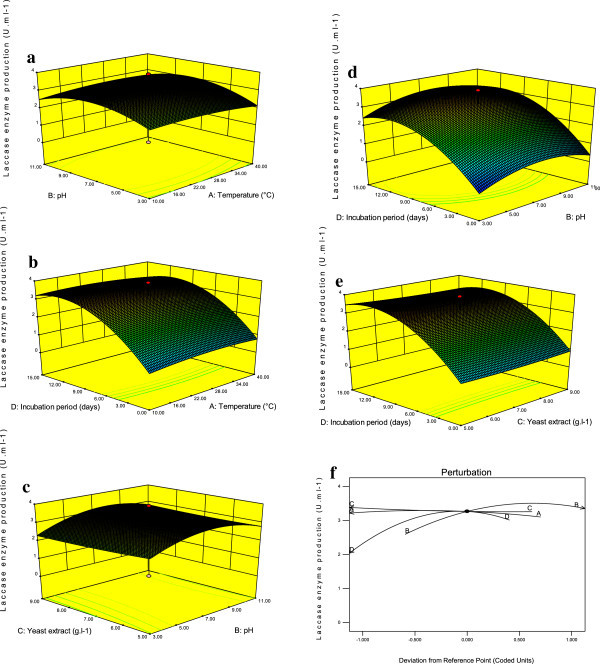
**Interactive effect of the culture conditions (a) effect of the pH and temperature, (b) effect of incubation period and temperature, (c) effect of yeast extract and pH, (d) effect of the incubation period and pH, (e) effect of the incubation period and yeast extract on laccase production, and (f) optimization of the laccase production of perturbation plot.**

The confirmation of the experimental setup for the laccase production was attributed by using normal probability, predicted response of analysis (Figure 3a-b). The regression analysis of the optimization study indicated that the model terms, X_1_, X_2_, X_4_, X_2_^2^, and X_4_^2^ were significant on laccase production by *Hypocrea lixii* (P <0.05) (Table 3 and Figure 4a-e). These results indicated that temperature, pH, incubation period had the direct association with laccase production. Lack of fit (0.92) also suggested that the obtained experimental data were of a good fit with the model (Table 3). The optimal conditions of the laccase production by the *Hypocrea lixii* was assessed by using a perturbation plot (Figure 4f). Statistically optimized conditions for the maximum laccase production was observed at the temperature of 29°C, pH of 5.3, yeast extract of 7.7 and incubation period of 12 days (Figure 4f).

### Molecular weight of laccase

**Figure 5:**
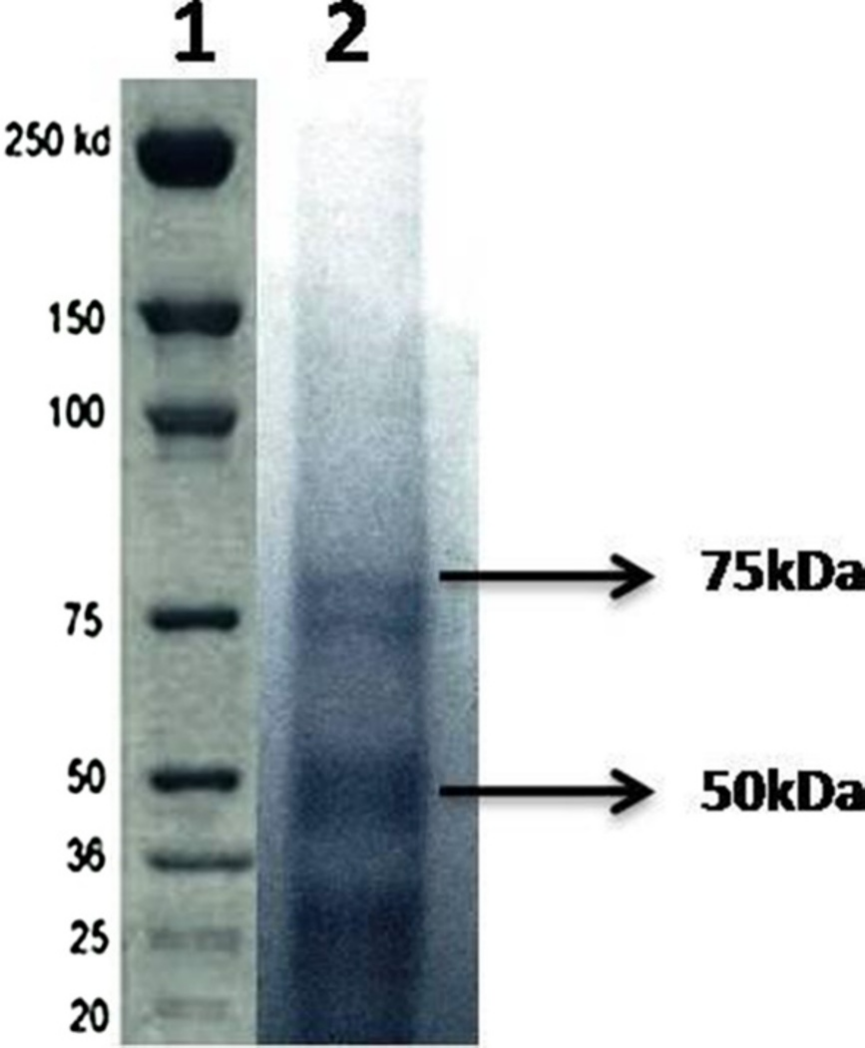
**SDS-PAGE patterns of purified laccaseof** ***H. lixii*** **culture extract.** (SDS-PAGE carried out using 5% polyacrylamide gel containing 0.1% SDS, and stained with 0.1% Coomassie brilliant blue R-250 after electrophoresis. Lane 1- Marker, molecular weight marker; lane 2 – Laccase from *H. lixii*).

The overall yield of the purification was around 58%, with a purification of 11.4 fold and a specific enzyme activity of 3.2 Umg^-1^. The partially purified enzyme showed a single band after SDS-PAGE (Figure 5). The molecular mass of purified laccases was found to be approximately 50 kDa and 75 kDa, as determined by SDS-PAGE.

### FT-IR (Fourier Transform Infra Red) studies

**Figure 6:**
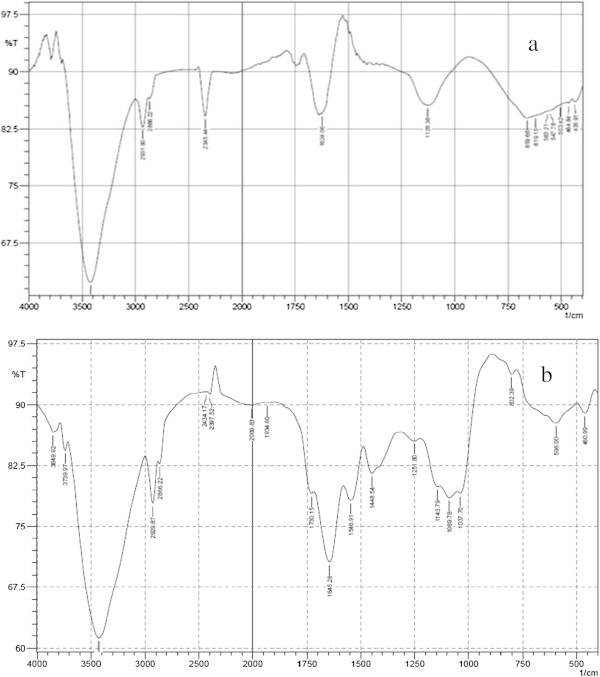
**FT-IR representation of dye degradation (a) before dye degradation (b) after dye degradation.**

In order to confirm the degradation of malachite green by *Hypocrea lixii*, the dye solution was subjected to FT-IR spectral analysis, before and after degradation. The FT-IR spectra of degraded dye and undegraded dye are shown in Figure 6a and b. FT-IR spectra of undegraded dye showed the specific peaks in a range between 1500 and 500 cm^-1^ for the mono and para-di substituted benzene rings. The peaks between 400 and 550 cm^-1^ represented the presence of sulphide groups and bromine groups. Also the peak at 1128.36 cm^-1^for the C-N stretching vibrations and peak at 2931 cm^-1^for C-H stretching of asymmetric -CH_3_group provided the perception of structure of malachite green. The degraded dye revealed an overall reduction in the spectra and both mono and para-di substituted benzene rings, sulphates and -CH_3_, O-H, N-H, and C ≡ N groups. The peak reduction was attributed to the cleavage of synthetic dye bonds. The dye got completely degraded after 10 days of incubation.

### Scanning electron microscopic (SEM) studies

**Figure 7:**
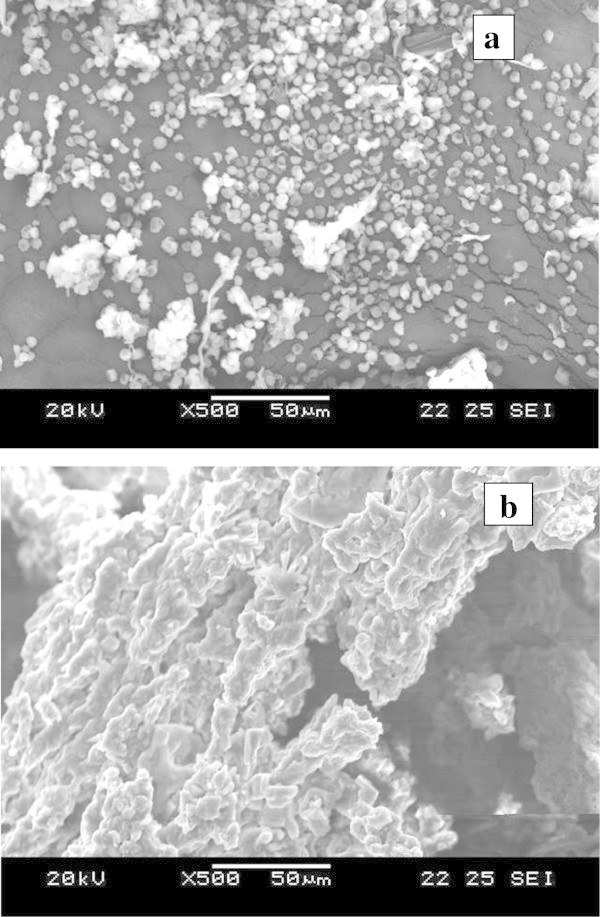
**Scanning electron microscopic representation of the dye degradation (a) Control untreated** ***Hypocrea lixii*** **spores (50 μm), (b) dye treated** ***Hypocrea lixii*** **spores (50 μm).**

Purpose of knowing the changes before and after accumulation of the dye in surface area of *Hypocrea lixii* biomass was studied by using SEM (Figure 7a-b). Significant changes were observed on the surface of microbial biomass after dye accumulation by *Hypocrea lixii* (Figure 7b). In the non- dye treated control, fungal spores were distinct, but in dye treated, the fungal spores were not clearly visible.

### Biotoxicity

**Figure 8:**
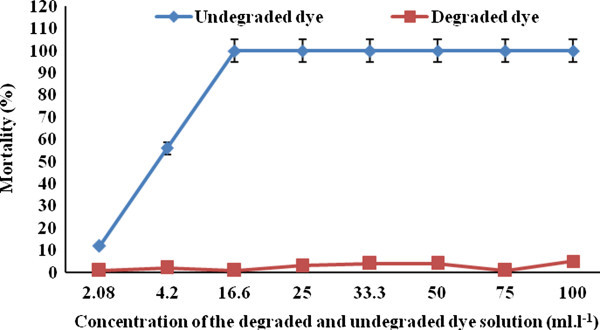
**Effect of toxicity of degraded and undegraded dye solution on** ***Artemia salina*** **culture.**

The toxicity of degraded and undegraded dye solutions was tested with *Artemia salina* and the results are presented in Figure 8. The mortality was observed 100% in the undegraded dye solution as against only 2-5% in degraded dye solution (Figure 8).

## Discussion

The present work showed the significant effect of the marine fungal strains *Hypocrea lixii* on dye degradation and laccase production (Figure 1a and b). This finds support of earlier workers who have demonstrated similar finding of dye degradation with spore forming fungal strains of the *Trichoderma harzianum*/*Hypocrea lixii* and *Aspergillus flavus, Fusarium oxysporum* and *F. moniliforme* for the textile dye degradation and enzyme production (Raju et al. 2007; Rajalakshmi and Sudha 2011; Kathiresan et al. 2011; Saravanakumar and Kathiresan 2012). *Trichoderma harzianum*/*Hypocrea lixii* (TSK8) has been improved mangroves plants growth (Saravanakumar et al. 2013) Laccase producing micro organisms are potent source for the degradation of the textile dye (Tavares et al. 2008). Dye decolourization by fungi during growth on solid medium has been widely employed to identify the ligninolytic potential and potential degradation of phenolic compounds (Wesenberg et al. 2003; Rajalakshmi and Sudha 2011). The laccase produced by *Hypocrea lixii* exhibited the molecular weight of the 50 and 75 kDa, similar to those reported for laccases produced by *Aspergillus* sp. (Youshuang et al. 2011).

The present study achieved 89% degradation of synthetic dye Malachite Green by *Hypocrea lixii* under the optimal conditions of temperature 30°C, pH of 5.8, yeast extract 5.81 mg.l^-1^ at incubation period of 10 days (Figure 2f). The dye degradation was significant with increased laccase in the solution. In the present study the maximum activity of laccase was observed on the 12^th^day and confirmed that 100% of dye was removed by laccase produced by *Hypocrea lixii* on the 12^th^ day of the degradation process. This report is in conformity with previous researchers (Ambrosio and Takaki 2004; Machii et al. 2004).

FTIR spectra confirmed the degradation of Malachite Green in the solution, as evident by the appearance of some new peaks and absence of important peaks required for structural integrity of the dyes. In the case of UV–vis absorption spectra, there was a complete disappearance of major visible light absorbance peaks (Figures 1b and 6a,b). A similar report has been made with methyl red degradation by *Sphingomonas paucimobilis* (Ayed et al. 2011). Scanning electron microscopic observations also confirmed the accumulation of the malachite green on the surface of the fungal biomass. The degraded dye showed low toxicity to artemia larvae. Thus the dye after degradation by *Hypocrea lixii*, if discharged to aquatic environments, may not cause any damage to the aquatic animals. Thus the laccase producing *Hypocrea lixii* is a promising fungal strain for degradation of synthetic dye.
